# Tuberculosis in Chickens

**Published:** 1896-06

**Authors:** T. A. Bray

**Affiliations:** Kansas City, Mo.


### TUBERCULOSIS IN CHICKENS.

By T. A. BRAY, D.V.S.,
KANSAS CITY, MO.

It is not generally known that chickens have tuberculosis, but it is a fact nevertheless, as there is ample proof of it.

A few weeks ago one of the employés in a Kansas City abattoir asked a government inspector what those little pimples were on a chicken he was packing for shipment. It did not take the doctor long to determine what he had before him. It was a well-developed case of tuberculosis. The doctor promptly condemned the carcass.

Last summer my attention was called to a number of chickens that were sick. The owner partly made his living by raising chickens for the market, having about one hundred on hand most of the time. After looking the chickens over, and getting all the history possible, I concluded there was an outbreak of some lung-trouble, and told the owner if any should die I would like to hold a post-mortem.

As I did not receive any word about any dead chickens, and having some spare time, I dropped in to see the sick ones. It was in the morning, and on arriving at the chicken-farm I noticed a man coughing and expectorating a mixture containing blood. The spring chickens were around him pretty thick, and when he expectorated they would devour it with a relish. I didn't have to ask the man what his ailment was, as he plainly showed it; but thought it no harm to ask him a few questions.

He informed me that his name was Johnson, and that he lived next door to the owner of the chickens, and as there was no division fence the chickens had the privilege of both yards. Every morning that he felt able he would venture into the yard, and usually had a coughing-spell, and expectorated a great deal. The chickens were always on hand to pick up what he coughed up. Mr. Johnson has had a number of brothers and sisters die from tuberculosis, so that it is quite general in his family. His present condition is such that he cannot get out during this weather, and is growing weaker as his case progresses.

By the owner's consent, I killed one of the chickens, and on post-mortem found a case of generalized tuberculosis. I also took one of the sick birds to the Kansas City Veterinary College, where a post-mortem was held. They also found a generalized case of tuberculosis.

The poultry farm lost a large percentage of their young chickens, and would have probably lost more if they had not been disposed of before showing advanced symptoms of the disease. The old chickens did not seem to take the disease, as the young birds were very quick in picking up what was expectorated, the old ones not getting a chance at the expectorated germs.

*Symptoms.* Standing about in a sleepy state; breathing accelerated. Other chickens picking at sick ones. Frequent coughing, which brings up a discharge from the lungs, and in the advanced stages this discharge can be found on the feathers of the back where the bird rests its head during the time it sleeps, the cough frequently disturbing its rest. This discharge dries on the feathers, and there is a great deal of it if the case has been long standing. The chicken becomes very much emaciated as the disease progresses.

*Post-mortem.* Liver, lungs, and walls of thorax showed evidence of tuberculosis, which was quite plain to the naked eye.

*Conclusion.* Chickens, as we all know, are extensively used for human food, and should be inspected as well as other food-products. Some of our abattoirs slaughter poultry on a large scale.

